# Porcine Skeletal Muscle-Specific lncRNA-ssc.37456 Regulates Myoblast Proliferation and Differentiation

**DOI:** 10.3390/ani16030361

**Published:** 2026-01-23

**Authors:** Xia He, Yangshuo Hu, Yangli Pei, Yilong Yao, Shen Liu

**Affiliations:** 1 School of Animal Science and Technology, Foshan University, Foshan 528225, China; 13310479911@163.com (X.H.); huyangshuo2024@163.com (Y.H.); peiyangli@163.com (Y.P.); 2 Agricultural Genomics Institute at Shenzhen, Chinese Academy of Agricultural Sciences, Shenzhen 518000, China; 3 Laboratory of Animal Nutritional Physiology and Metabolic Process, Key Laboratory of Agroecological Processes in Subtropical Region, National Engineering Laboratory for Pollution Control and Waste Utilization in Livestock and Poultry Production, Institute of Subtropical Agriculture, Chinese Academy of Sciences, Changsha 410125, China

**Keywords:** lncRNA-ssc.37456, muscle specificity, porcine primary myoblast proliferation and differentiation

## Abstract

Long-chain non-coding RNAs (lncRNAs) play important regulatory roles in the growth and development of skeletal muscle, but systematic identification and functional studies of lncRNAs related to porcine skeletal muscle development remain limited. Based on a previously constructed panoramic map of porcine skeletal muscle lncRNAs, lncRNA-ssc.37456 was identified as differentially expressed in porcine skeletal muscle before and after birth. Its function and potential mechanisms were investigated using a porcine skeletal muscle regeneration model, a primary skeletal muscle cell differentiation model, and knockdown and overexpression experiments in vitro. lncRNA-ssc.37456 was upregulated on day 7 of regeneration, with expression positively correlated with the muscle differentiation marker *MYHC* and negatively correlated with the proliferation marker *PAX7*. During differentiation of porcine primary myoblasts, expression continuously increased, peaking on day 4. Knockdown of lncRNA-ssc.37456 by small interfering RNA (siRNA) significantly increased cell proliferation, upregulated mRNA and protein levels of proliferation-related genes *KI67* and *PCNA*, and increased the proportion of EdU-positive cells. Conversely, expression of differentiation-related genes *MYOG* and *MYHC* decreased, and immunofluorescence analysis revealed reduced myotube formation and differentiation index. Overexpression of lncRNA-ssc.37456 promoted differentiation and inhibited proliferation, showing effects opposite to those observed in knockdown experiments. Nucleocytoplasmic fractionation indicated predominant cytoplasmic localization, suggesting potential function through a ceRNA mechanism. An interaction network with miRNAs was constructed based on the miRDB database, indicating a potential miRNA “sponge” regulatory mechanism. These results indicate that lncRNA-ssc.37456 participates in porcine skeletal muscle development by regulating the transition of muscle cells from proliferation to differentiation, providing molecular insights and potential targets for muscle biology research and the molecular breeding of growth traits.

## Simple Summary

The long non-coding RNA ssc.37456 exhibits dynamic expression changes during pig skeletal muscle development. Knockdown of ssc.37456 in a pig muscle injury model and in primary muscle cells increased cell proliferation but impaired myofiber formation, whereas overexpression suppressed proliferation and promoted muscle maturation. Subcellular localization analysis indicated that ssc.37456 is predominantly cytoplasmic, suggesting potential interactions with small regulatory RNAs to modulate gene expression. These results identify ssc.37456 as a regulator of pig muscle growth and indicate its potential utility for improving growth performance and meat production through molecular breeding.

## 1. Introduction

Skeletal muscle is one of the most abundant and metabolically active tissues in mammals, accounting for approximately 40% of body mass [1]. It not only constitutes the main component of the locomotor system but also plays a central role in maintaining systemic energy homeostasis, glucose utilization, lipid oxidation, and thermoregulation [2]. In pigs, skeletal muscle development and composition directly determine carcass lean percentage, meat yield, and overall pork quality, making it a key biological determinant of both production efficiency and economic value [3,4]. The development of skeletal muscle is a highly orchestrated, spatiotemporally regulated process that involves sequential stages of myogenic progenitor activation, proliferation, differentiation, fusion, and fiber maturation, coordinated by genetic, hormonal, nutritional, and epigenetic factors [5,6,7,8]. During the fetal period, muscle growth is mainly achieved through the proliferation and fusion of myogenic cells to establish fiber number, while postnatal growth primarily depends on hypertrophy of existing fibers [9,10]. Therefore, the perinatal stage represents a critical window for muscle development, as the degree of myogenesis during this period not only determines postnatal growth potential and lean meat yield but also affects meat quality traits such as tenderness, water-holding capacity, and intramuscular fat content [11,12]. With the rapid progress of molecular breeding and precision selection technologies, identifying key regulators and molecular mechanisms governing skeletal muscle development has become crucial for improving pork yield and quality in modern breeding programs [13,14].

Long non-coding RNAs (lncRNAs) are RNA transcripts longer than 200 nucleotides that lack protein-coding potential but perform diverse regulatory functions [15,16,17]. They modulate gene expression through multiple mechanisms, including chromatin remodeling, transcriptional activation or repression, miRNA sponging via the ceRNA mechanism, and post-transcriptional regulation of mRNA stability and translation [18,19,20]. Over the past decade, lncRNAs have emerged as key regulators of embryogenesis, stem cell fate determination, tissue regeneration, and metabolic homeostasis [21,22]. In muscle biology, several well-characterized lncRNAs, such as linc-MD1, H19, Neat1, and LncMyoD, have been shown to regulate myoblast proliferation and differentiation, thereby contributing to myofiber formation and regeneration [23,24,25,26]. These findings underscore the spatial and temporal specificity of lncRNA expression and the complexity of their regulatory networks in myogenesis. However, relatively low expression levels, limited sequence conservation, and incomplete annotation of porcine lncRNAs continue to hinder their functional characterization and potential application in molecular breeding.

In this study, we systematically screened skeletal muscle transcriptome data from multiple pig breeds (Meishan, Wuzhishan, Yorkshire, and Tibetan) at different developmental stages (day 0, day 180, and day 300) and identified a novel lncRNA, ssc.37456, that was differentially expressed between prenatal and postnatal stages [27]. During the key myofiber formation period (D9–D80), ssc.37456 exhibited significantly higher expression in Tongcheng pigs than in Landrace pigs. Notably, ssc.37456 was found to be specifically enriched in skeletal muscle tissues and predominantly localized in the cytoplasm of primary porcine myoblasts, suggesting its potential role in post-transcriptional regulation during muscle development. Functional studies demonstrated that ssc.37456 promotes myogenic differentiation while suppressing myoblast proliferation. Moreover, its expression dynamically fluctuated during muscle injury-induced regeneration, implying an involvement in regenerative remodeling. Collectively, our findings identify ssc.37456 as a novel lncRNA associated with porcine skeletal muscle development and reveal its potential role in regulating myogenic cell fate. This work provides new insights into the epigenetic regulatory network underlying muscle growth and offers a promising molecular target for lncRNA-based precision breeding in pigs.

## 2. Materials and Methods

### 2.1. Establishment of the Skeletal Muscle Regeneration Model

One-month-old male Tibetan pigs were used to establish a skeletal muscle regeneration model. All animal procedures were conducted in accordance with the Guidelines for the Care and Use of Laboratory Animals and were approved by the Institutional Animal Care and Use Committee (IACUC) of the Biomedical Research Ethics Committee of the Agricultural Genomics Institute at Shenzhen, Chinese Academy of Agricultural Sciences (Approval No. [AGIS2020.04.17]). For induction of acute muscle injury, pigs were anesthetized, and 100 μL of 10 mM cardiotoxin (CTX; Sigma-Aldrich, St. Louis, MO, USA) was injected intramuscularly into the mid-belly region of the biceps femoris muscle at the designated site using a sterile syringe. Control muscles (day 0) were collected prior to CTX injection. Injured muscle tissues were harvested at 0, 3, 5, 7, and 14 days post-injection, immediately snap-frozen in liquid nitrogen or fixed as required, and processed for subsequent molecular and histological analyses.

### 2.2. Primer Design

Primers were designed using Primer Premier 5 software. Small interfering RNAs (siRNAs) and their negative controls were synthesized by GenePharma Co., Ltd. (Shanghai, China). The sequences of all primers are listed in Table 1, and those of siRNAs are presented in Table 2.

### 2.3. Transcriptome Data of Landrace and Tongcheng Pigs

Transcriptome datasets from Landrace and Tongcheng pigs across 27 developmental time points were obtained from our previously published study [27]. These data were used for all transcriptomic analyses in the current study. Details regarding sample collection, RNA extraction, sequencing protocols, and quality control have been described in the original publication.

### 2.4. Plasmid Construction

The full-length transcript of lncRNA-ssc.37456 was amplified by PCR using forward and reverse primers containing BamHI and XhoI restriction enzyme sites. PCR amplification was performed using ApexHF HS DNA Polymerase FS Master Mix (AG12202, Accurate Biotechnology, Changsha, Hunan, China). The amplified fragment was subsequently ligated into the pcDNA3.1 expression vector (Invitrogen, Carlsbad, CA, USA) to generate the overexpression construct pcDNA3.1-lncRNA-ssc.37456, while the empty pcDNA3.1 vector served as a negative control. All recombinant plasmids were verified by Sanger sequencing. In addition, luciferase reporter plasmids containing the wild-type (WT) or mutant (MUT) miRNA-binding sites of lncRNA-ssc.37456 were constructed using the pmirGLO vector (Promega, Madison, WI, USA). All plasmids were synthesized and sequence-verified by GENECREATE Biotechnology Co., Ltd. (Wuhan, China).

### 2.5. Isolation and Culture of Primary Porcine Skeletal Muscle Satellite Cells

Primary skeletal muscle satellite cells were isolated from the hindlimb muscles of male Tibetan piglets younger than one week, as previously described [19]. Briefly, skeletal muscle tissue was finely minced and digested with collagenase type II (300 U/mL; Gibco, Grand Island, NY, USA) at 37 °C for 30 min in a shaking water bath. The digestion was terminated by adding high-glucose DMEM (Gibco) supplemented with 10% fetal bovine serum (FBS; Gibco) and 2% penicillin-streptomycin. The cell suspension was sequentially filtered through 100 μm, 70 μm, and 40 μm cell strainers to remove debris and connective tissue. The filtrate was centrifuged and resuspended in RPMI-1640 medium. Purified primary myoblasts were seeded on Matrigel-coated (BD Biosciences, San Jose, CA, USA) plates and cultured in growth medium until reaching ~90% confluence. For differentiation induction, the medium was replaced with DMEM containing 8% horse serum (Gibco), and cells were cultured for the indicated periods.

### 2.6. Cell Transfection

Transient transfections were performed using Lipofectamine RNAiMAX (Cat. No. 13778150 Invitrogen, Carlsbad, CA, USA) for siRNA delivery and Lipofectamine 2000 (Cat. No. 11668019 Invitrogen, Carlsbad, CA, USA) for plasmid transfection, following the manufacturer’s instructions. Cells were transfected at approximately 60–70% confluence and harvested 24–48 h post-transfection for downstream analyses.

### 2.7. Quantitative Real-Time PCR (qRT-PCR)

Total RNA from tissues or cultured cells was extracted using TRIzol reagent (TaKaRa, Kusatsu, Shiga, Japan) according to the manufacturer’s instructions. RNA concentration and purity were assessed using a NanoDrop 2000 spectrophotometer (Thermo Fisher Scientific, Waltham, MA, USA), and only samples with A260/A280 ratios between 1.8 and 2.1 were used for subsequent analyses. RNA integrity was further evaluated by agarose gel electrophoresis.

For nuclear and cytoplasmic RNA isolation, the Cytoplasmic & Nuclear RNA Purification Kit (TaKaRa, Kusatsu, Shiga, Japan) was used following the manufacturer’s protocol. For mRNA and lncRNA detection, cDNA was synthesized using the HiScript III 1st Strand cDNA Synthesis Kit (Vazyme, Nanjing, China), which includes a genomic DNA removal step. Quantitative real-time PCR was performed using ChamQ SYBR qRT-PCR Master Mix (Vazyme, Nanjing, China) on a QuantStudio 6 Flex Real-Time PCR System (Applied Biosystems, Foster City, CA, USA).

For miRNA analysis, reverse transcription was conducted using the miRNA 1st Strand cDNA Synthesis Kit (Vazyme, Nanjing, China), followed by qRT-PCR using the miRNA Universal SYBR qRT-PCR Master Mix (Vazyme, Nanjing, China). Relative gene expression levels were normalized to *GAPDH* (for mRNA and lncRNA) or U6 (for miRNA) and calculated using the 2^−ΔΔCt^ method.

For one-step RT-PCR analysis, the HiScript II One-Step RT-PCR Kit (Vazyme, P611-01) was used according to the manufacturer’s instructions.

### 2.8. 5-Ethynyl-2′-Deoxyuridine (EdU) Assay

Cell proliferation was assessed using a BeyoClick™ EdU Cell Proliferation Kit (Beyotime, Shanghai, China). Cells were seeded in 12-well plates and transfected at 70–80% confluence. After 48 h, cells were incubated with 20 μM EdU for 2 h, fixed with 4% paraformaldehyde for 15–30 min, and permeabilized with 0.05% Triton X-100 for 10–15 min. The Click-iT reaction cocktail was prepared according to the manufacturer’s instructions and added to each well for 30 min in the dark. Nuclei were counterstained with DAPI (1:1000), and EdU-positive cells were visualized using a fluorescence microscope (Olympus IX73, Tokyo, Japan). The proliferation rate was quantified as the ratio of EdU-positive nuclei to total nuclei using ImageJ (ImageJ v1.53 (NIH, Bethesda, MD, USA)).

### 2.9. Immunofluorescence Staining

Primary porcine myoblasts were cultured on 12-well plates and induced to differentiate for 48 h after transfection. Cells or tissue sections were fixed with 4% paraformaldehyde for 20 min, washed with PBS, and permeabilized with 0.05% Triton X-100 for 15 min. After blocking with 5% bovine serum albumin (BSA) for 1 h at room temperature, samples were incubated with the primary antibody anti-MYHC (3 μg/mL; DSHB, Iowa City, IA, USA) overnight at 4 °C. Secondary antibodies, including FITC- or Cy3-conjugated donkey anti-rabbit/mouse IgG (Servicebio, Wuhan, China), were applied for 1 h at room temperature in the dark. Nuclei were counterstained with DAPI (1:1000), and images were captured using an Olympus IX73 fluorescence microscope. Myotube formation and MYHC fluorescence intensity were analyzed using ImageJ software.

### 2.10. Subcellular Fractionation of Nuclear and Cytoplasmic RNA

To determine the subcellular localization of lncRNA-ssc.37456, cytoplasmic and nuclear RNA fractions were isolated using the PARIS™ Kit (Thermo Fisher Scientific, USA) according to the manufacturer’s instructions. Briefly, cells were lysed in ice-cold cell fractionation buffer and centrifuged at 500× *g* for 5 min at 4 °C. The supernatant and pellet were collected as the cytoplasmic and nuclear fractions, respectively. RNA was then extracted from each fraction following the manufacturer’s protocol and subjected to subsequent qRT-PCR analysis.

### 2.11. Hematoxylin and Eosin (H&E) Staining

Skeletal muscle tissues were fixed in 4% paraformaldehyde for 24–36 h, dehydrated, embedded in paraffin, and sectioned at 5 μm thickness. Sections were subjected to routine H&E staining, dehydration, clearing, and coverslipping with neutral resin. Histological morphology was observed under a light microscope (Olympus, Tokyo, Japan).

### 2.12. Western Blotting

Cells were seeded in 6-well plates and harvested 48 h after transfection for protein extraction using a protein lysis buffer (78501, Thermo Scientific™, Waltham, MA, USA). Western blotting was performed following standard procedures. Proteins were transferred onto PVDF membranes (24937-79-9, Millipore, Billerica, MA, USA), which were then blocked with skim milk powder (D8340, Solarbio, Beijing, China) for 2 h. The following primary antibodies were used: PCNA (1:1000, ab29, Abcam, Cambridge, UK), Ki67 (1:1000, ab16667, Abcam, Cambridge, UK), MyOG (1:1000, DF8273, Affinity, Changzhou, Jiangsu, China), MYHC (1:1000, MF20, DSHB, Iowa City, IA, USA), and GAPDH (1:1000, 5174, Cell Signaling Technology, Danvers, MA, USA). Membranes were incubated with primary antibodies for 3 h at room temperature or overnight at 4 °C, followed by incubation with HRP-conjugated secondary antibodies (goat anti-rabbit IgG H&L, ab6721 and ab205719, Cambridge, UK Abcam) for 2 h. Membranes were washed three times with TBST (10 min each). Signals were detected using a Tanon imaging system (3500, Shanghai, China). Band intensities were quantified using ImageJ software.

### 2.13. Statistical Analysis

Statistical analyses were performed using GraphPad Prism 8 (GraphPad Software, San Diego, CA, USA). Data are presented as mean ± standard deviation (SD). Differences between two groups were analyzed using a two-tailed Student’s *t*-test. Statistical significance was set at *p* < 0.05 (ns, not significant; *p* < 0.05, * *p* < 0.01). All experiments were performed in at least three independent biological replicates.

## 3. Results

### 3.1. Identification of Skeletal Muscle-Specific lncRNAs and Their Developmental Expression Dynamics

**Figure 1:**
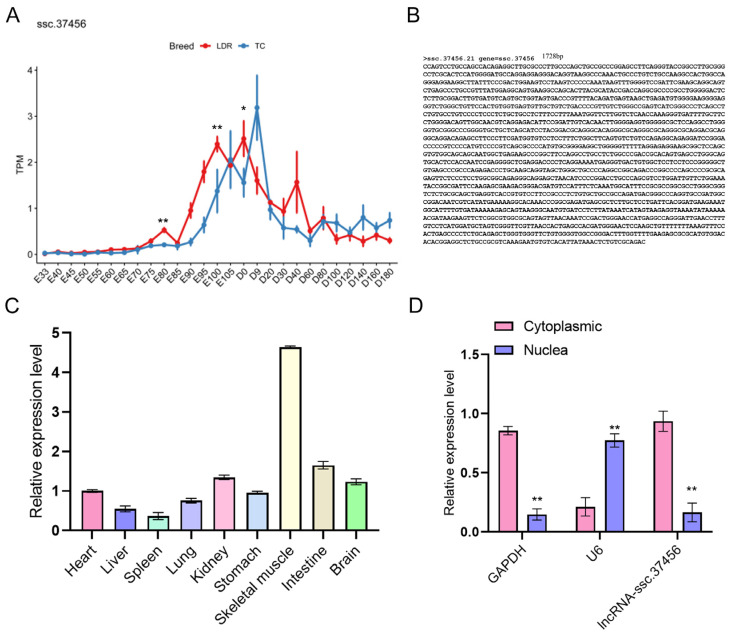
The expression patterns of lncRNA-ssc.37456. (**A**) Expression dynamics of lncRNA-ssc.37456 (TPM) in longissimus dorsi muscle of Landrace (LDR, red) and Tongcheng (TC, blue) pigs at embryonic (E33–E105) and postnatal (D9–D180) developmental stages. (**B**) Nucleotide sequence of lncRNA-ssc.37456 (1728 bp). (**C**) qRT-PCR analysis of lncRNA-ssc.37456 expression in different porcine tissues, including heart, liver, spleen, lung, kidney, stomach, skeletal muscle, intestine and brain. (**D**) Subcellular localization of lncRNA-ssc.37456 in primary porcine skeletal muscle cells determined by qRT-PCR of nuclear and cytoplasmic fractions. *GAPDH* and U6 serve as cytoplasmic and nuclear controls, respectively. All quantitative data are presented as mean ± standard deviation (SD); * *p* < 0.05, ** *p* < 0.01.

Talogue lncRNAs potentially involved in porcine myogenesis, a strand-specific RNA-seq atlas comprising 246 samples from diverse tissues and developmental stages (NCBI BioProject PRJNA612825) was assembled. Analysis detected 26,279 expressed lncRNA transcripts, including 10,173 previously unannotated loci. Tissue specificity profiling identified 150 tissue-restricted lncRNAs, of which 63 exhibited preferential expression in skeletal muscle, suggesting potential roles in muscle development. Comparative transcriptomic analysis of longissimus dorsi muscle from Tongcheng and Landrace pigs across embryonic and postnatal stages revealed 12 skeletal muscle–specific lncRNAs with pronounced developmental dynamics. Among these, the lncRNA-ssc.37456 is a transcript on chromosome 2 with a length of 2,441,728 nt; its expression shows a temporal pattern with a sharp increase during late embryogenesis, a peak around early postnatal growth, and a subsequent decline toward adulthood, and during myofiber formation its expression is consistently higher in Tongcheng pigs than in Landrace pigs. (Figure 1A,B). qRT-PCR analysis across multiple postnatal tissues indicated that ssc.37456 is highly enriched in skeletal muscle relative to heart, liver, spleen, lung, kidney, stomach, intestine, and brain (Figure 1C). Subcellular fractionation of primary porcine skeletal muscle cells revealed that ssc.37456 is predominantly localized in the cytoplasm, in contrast to the nuclear-enriched control U6 and the cytoplasmic *GAPDH* mRNA (Figure 1D). These data identify ssc.37456 as a skeletal muscle–enriched, developmentally regulated lncRNA with breed-dependent expression and predominant cytoplasmic localization, consistent with a potential role in post-transcriptional regulation during myogenesis.

### 3.2. LncRNA-ssc.37456 Is Upregulated During Muscle Regeneration and Myoblast Differentiation

**Figure 2:**
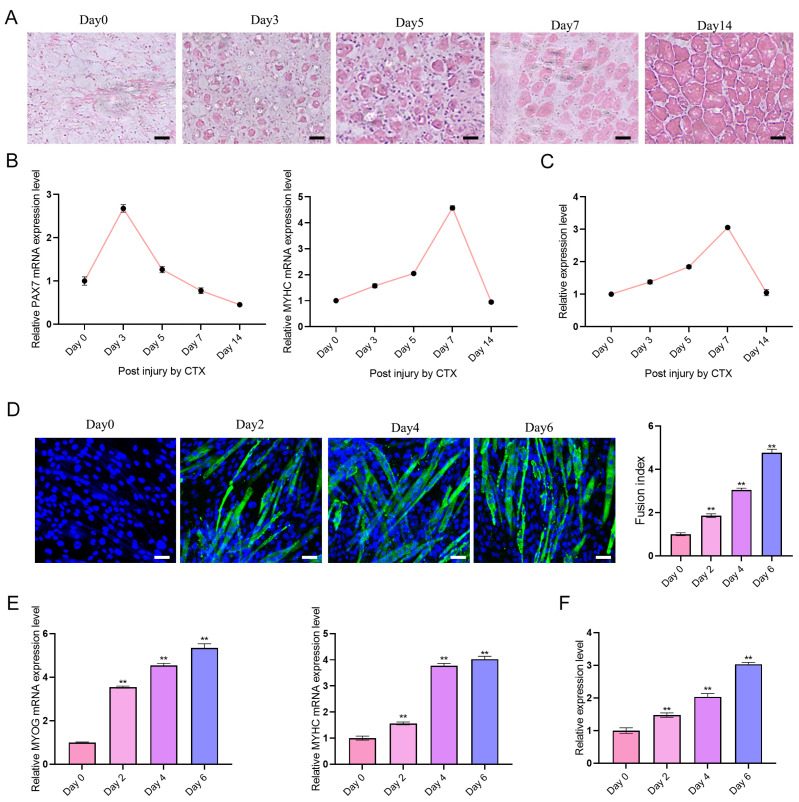
Expression characteristics of lncRNA ssc.37456 during skeletal muscle injury repair and satellite cell differentiation. (**A**) H&E staining showing histological changes in the tibialis anterior muscle of Tibetan pigs at different time points (0, 3, 5, 7, and 14 days) after CTX-induced injury. Scale bar: 50 µm. (**B**) qRT-PCR analysis of *PAX7* (left) and *MYHC* (right) mRNA expression in CTX-treated skeletal muscle at the indicated time points. (**C**) qRT-PCR analysis of lncRNA-ssc.37456 expression in CTX-treated skeletal muscle during regeneration. (**D**) Immunofluorescence staining of MYHC (green) and DAPI (blue) showing myotube formation of porcine muscle satellite cells at different days of in vitro differentiation (0, 2, 4 and 6 days). The **right panel** shows quantification of the fusion index. Scale bar: 100 µm. (**E**) qRT-PCR analysis of *MYOG* (**left**) and *MYHC* (**right**) mRNA expression in muscle satellite cells during in vitro differentiation. (**F**) qRT-PCR analysis of lncRNA- ssc.37456 expression in muscle satellite cells at different stages of in vitro differentiation. All data are presented as mean ± standard deviation (SD); ** *p* < 0.01.

Expression dynamics of lncRNA-ssc.37456 were examined during skeletal muscle regeneration in vivo using a CTX-induced injury model in one-month-old Tibetan pigs. H&E staining revealed temporal progression of regeneration: uninjured, well-organized myofibers at Day 0; extensive myofiber necrosis and inflammatory cell infiltration at Day 3; appearance of centrally nucleated regenerating fibers at Day 5; numerous newly regenerated myofibers by Day 7; and near-complete restoration of normal morphology by Day 14 (Figure 2A). qRT-PCR analysis showed that the myoblast marker *PAX7* peaked at Day 3 and then declined, whereas the differentiation marker *MYHC* reached maximal expression at Day 7 (Figure 2B). LncRNA-ssc.37456 expression increased progressively during regeneration, peaking at Day 7, paralleling the pattern of *MYHC* (Figure 2C). During in vitro differentiation of primary porcine skeletal muscle satellite cells, immunofluorescence staining for *MYHC* showed a time-dependent increase in *MYHC*-positive myotubes from Day 2 to Day 6, accompanied by an increase in the fusion index (Figure 2D). qRT-PCR analysis revealed upregulation of the myogenic regulator *MYOG* from Day 2 through Day 6, while *MYHC* mRNA levels increased continuously over the same period (Figure 2E). LncRNA-ssc.37456 expression also rose progressively during differentiation, reaching the highest level at Day 6 (Figure 2F). These results indicate that lncRNA-ssc.37456 is induced during the differentiation and regeneration phases of myogenesis, with expression dynamics closely matching those of key myogenic markers.

### 3.3. Knockdown of LncRNA-ssc.37456 Is Upregulated During Muscle Regeneration and Myoblast Differentiationimpairs Differentiation and Enhances Proliferation of Primary Myoblasts

**Figure 3:**
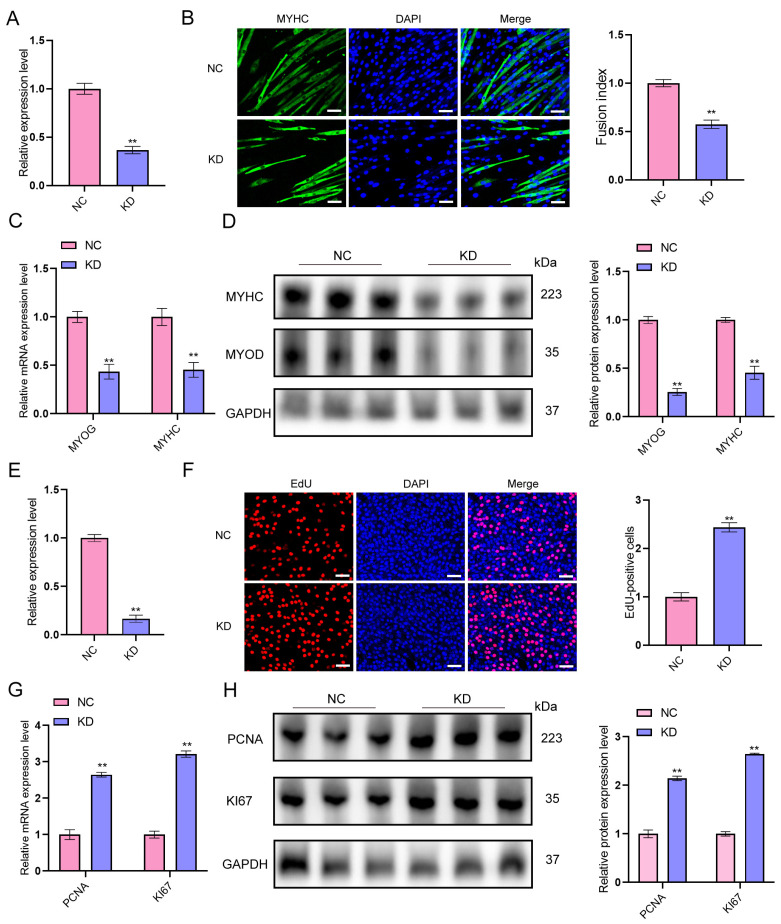
Effects of knockdown of lncRNA-ssc.37456 on muscle satellite cell differentiation and proliferation. (**A**) qRT-PCR analysis confirming the knockdown efficiency of lncRNA-ssc.37456 in the siRNA group (KD) compared with the negative control (NC). (**B**) Representative immuno-fluorescence images of MYHC (green) and nuclei (DAPI, blue) showing reduced MYHC-positive myotube formation in the KD group versus NC; quantification of the fusion index is shown on the right. Scale bar: 50 µm. (**C**) qRT-qRT-PCR analysis showing decreased mRNA levels of myogenic markers *MYOG* and *MYHC* in the KD group compared with NC. (**D**) Western blot analysis showing reduced protein levels of MYHC and MYOD in the KD group compared with NC; GAPDH was used as the loading control, and densitometric quantification is shown on the right. (**E**) qRT-PCR analysis confirming reduced expression of lncRNA-ssc.37456 in the KD group compared with NC. (**F**) EdU incorporation assay showing a higher proportion of EdU-positive cells in the KD group versus NC; quantification is shown on the right. Scale bar: 50 µm. (**G**) qRT-PCR analysis showing increased mRNA levels of proliferation markers *PCNA* and *Ki67* in the KD group compared with NC. (**H**) Western blot analysis showing increased protein levels of *PCNA* and *Ki67* in the KD group compared with NC; *GAPDH* was used as the loading control, and densitometric quantification is shown on the right. All bar graphs represent mean ± standard deviation (SD); ** *p* < 0.01.

To further investigate the functional role of lncRNA-ssc.37456 in myogenesis, siRNA-mediated knockdown was performed in primary porcine myoblasts (Figure 3A). Compared with NC cells, knockdown of lncRNA-ssc.37456 markedly impaired myogenic differentiation. Immunofluorescence staining for *MYHC* revealed fewer and shorter MYHC-positive myotubes in KD cultures, accompanied by a significant decrease in the fusion index relative to NC (Figure 3B). Consistently, qRT-PCR analysis showed that *MYOG* and *MYHC* mRNA levels were significantly lower in KD cells than in NC, and Western blotting confirmed reduced protein abundance of both *MYOG* and *MYHC* compared with NC (Figure 3C,D). In contrast, KD cells exhibited enhanced proliferation, EdU incorporation assays demonstrated a higher proportion of EdU-positive cells relative to NC (Figure 3E,F), and qRT-PCR and Western blot analyses showed elevated mRNA and protein levels of *PCNA* and *KI67* compared with NC (Figure 3G,H). These results indicate that, relative to NC, knockdown of ssc.37456 suppresses myogenic differentiation while promoting myoblast proliferation.

### 3.4. Overexpression of lncRNA-ssc.37456 Enhances Differentiation and Suppresses Proliferation

**Figure 4:**
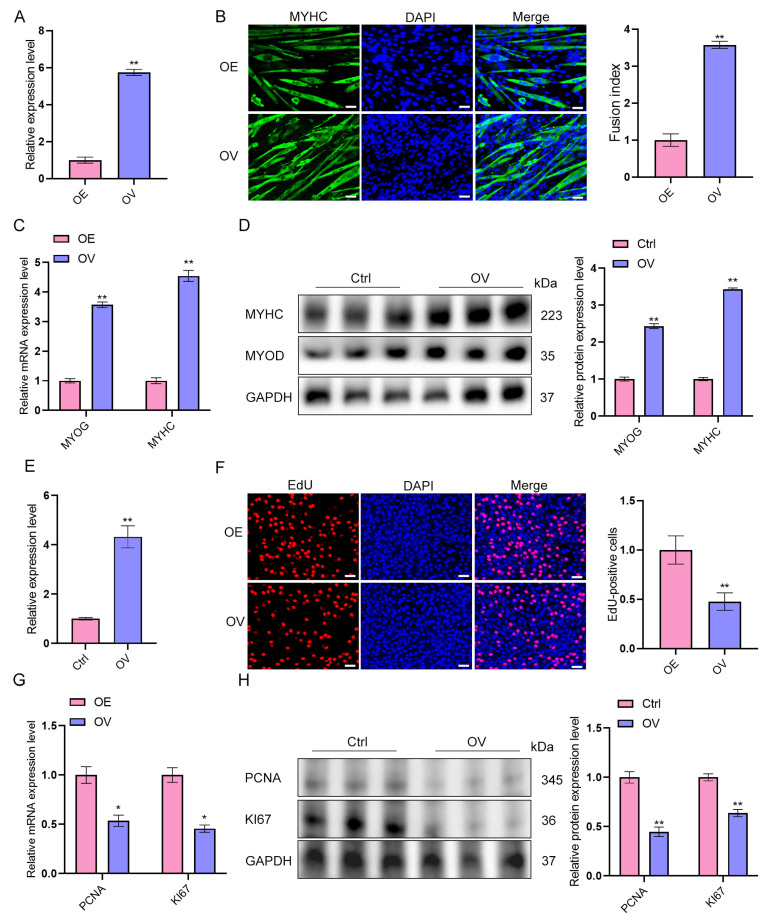
Effect of overexpression of lncRNA-ssc.37456 on differentiation and proliferation of porcine skeletal muscle satellite cells. (**A**) qRT-PCR analysis confirming the overexpression efficiency of lncRNA-ssc.37456 in the overexpression group (OV) compared with the vector control (Ctrl). (**B**) Representative immunofluorescence staining of MYHC (green) and nuclei (DAPI, blue) in Ctrl and OV cells. The fusion index is quantified on the right. Scale bar, 50 µm. (**C**) qRT-PCR analysis showing increased mRNA expression of myogenic marker genes *MYOG* and *MYHC* in OV compared with Ctrl. (**D**) Immunoblotting analysis showing increased protein levels of MYHC and MYOD in OV compared with Ctrl. GAPDH was used as a loading control. Densitometric quantification is shown on the right. (**E**) qRT-PCR analysis of lncRNA-ssc.37456 expression in OV compared with Ctrl (independent validation). (**F**) EdU incorporation assay showing a decreased proportion of EdU-positive cells in OV compared with Ctrl. Quantification is shown on the right. Scale bar, 50 µm. (**G**) RT–qPCR analysis showing reduced mRNA expression of proliferation markers *PCNA* and *MKI67* (*Ki67*) in OV compared with Ctrl. (**H**) Immunoblotting analysis showing reduced protein levels of PCNA and Ki67 in OV compared with Ctrl. GAPDH was used as a loading control. All data are presented as mean ± standard deviation (SD); * *p* < 0.05, ** *p* < 0.01.

To further investigate the functional role of lncRNA-ssc.37456 in myogenesis, overexpression (OE) was performed in primary porcine myoblasts. Compared with Ctrl cells, overexpression of lncRNA-ssc.37456 significantly promoted myogenic differentiation. Immunofluorescence staining for *MYHC* revealed more and longer MYHC-positive myotubes in OE cultures, accompanied by a significant increase in the fusion index relative to Ctrl (Figure 4A,B). Consistently, qRT-PCR analysis showed that *MYOG* and *MYHC* mRNA levels were significantly higher in OE cells than in Ctrl, and Western blotting confirmed increased protein abundance of both *MYOG* and *MYHC* compared with Ctrl (Figure 4C,D). In contrast, OE cells exhibited reduced proliferation: EdU incorporation assays demonstrated a lower proportion of EdU-positive cells relative to Ctrl (Figure 4E,F), and qRT-PCR and Western blot analyses showed decreased mRNA and protein levels of *PCNA* and *KI67* compared with Ctrl (Figure 4G,H). These results indicate that, relative to Ctrl, overexpression of ssc.37456 enhances myogenic differentiation while inhibiting myoblast proliferation.

### 3.5. Construction of the lncRNA-ssc.37456–miRNA Regulatory Network

**Figure 5:**
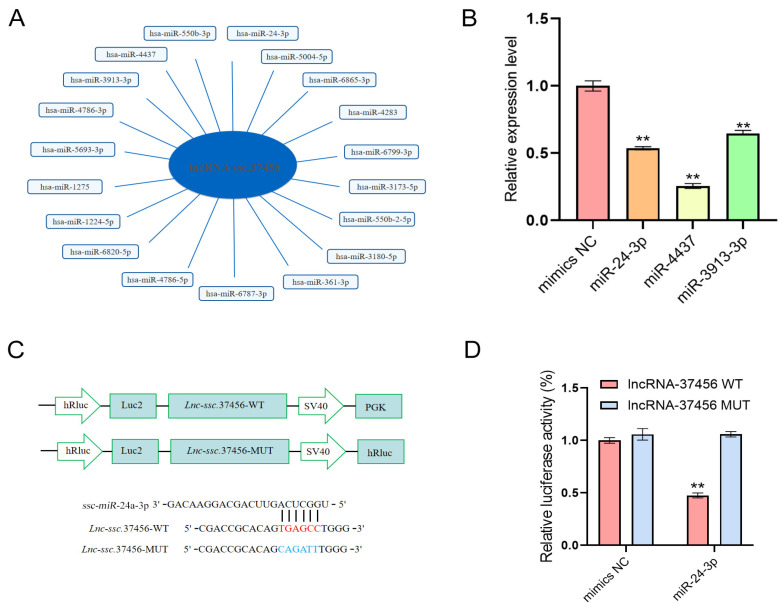
Sequence of lncRNA-ssc.37456 and its predicted miRNA interaction network. (**A**) Predicted interaction network of lncRNA-ssc.37456 (blue center node) with its potential target miRNA (light blue peripheral node). (**B**) q-PCR detection of the effect of miRNA on lncRNA-ssc.37456 expression. (**C**) Construction of luciferase reporter vectors for wild-type and mutant lncRNA-ssc.37456 containing miR-24a-3p. (**D**) Dual-luciferase assay to detect the binding of miR-24-3p to lncRNA-ssc.37456. All data are presented as mean ± standard deviation (SD); ** *p* < 0.01.

To investigate whether lncRNA-ssc.37456 functions as a competing endogenous RNA (ceRNA), miRNA binding sites were predicted using miRDB, identifying 19 candidate miRNAs, including miR-24-3p, miR-4437, and miR-3913-3p, all implicated in myogenesis and fiber type determination (Figure 5A). Overexpression of these miRNAs in vitro significantly reduced lncRNA-ssc.37456 levels (Figure 5B). Luciferase reporter assays further confirmed direct binding of miR-24-3p to lncRNA-ssc.37456, resulting in suppressed reporter activity (Figure 5C,D). These data provide mechanistic evidence supporting a ceRNA role for lncRNA-ssc.37456 in post-transcriptional regulation of myogenesis, laying the groundwork for downstream target validation.

## 4. Discussion

Long non-coding RNAs (lncRNAs) have emerged as pivotal regulators of skeletal muscle development, functioning at multiple regulatory levels to orchestrate myogenic lineage commitment, differentiation, and tissue remodeling [28,29,30,31]. Increasing evidence from humans and model organisms such as mice has identified numerous muscle-enriched lncRNAs, including linc-MD1, Dum, and LncMyoD, which control myogenesis through diverse molecular mechanisms—such as modulating transcription factor activity, acting as competing endogenous RNAs (ceRNAs), or recruiting chromatin remodeling complexes to muscle-specific loci [32,33,34,35,36,37]. Despite significant progress in model species, functional annotation of lncRNAs in livestock, particularly pigs, remains limited. Given that skeletal muscle growth and differentiation directly determine carcass composition and meat quality, the identification of muscle-specific lncRNAs in pigs is not only of biological interest but also of considerable economic importance. In our previous study, we constructed a comprehensive, strand-specific lncRNA expression atlas across 246 tissues and developmental stages in pigs, revealing 26,279 differentially expressed lncRNAs, of which 10,173 were previously unannotated [27]. Tissue-specific analysis identified 63 muscle-enriched lncRNAs, 43 of which were newly discovered, markedly expanding the catalog of porcine muscle-associated lncRNAs. Moreover, integration with eQTL and phenotypic association analyses linked several lncRNAs to key economic traits, such as average daily gain, carcass weight, and longissimus dorsi muscle area, suggesting their potential as molecular markers and regulatory candidates in genetic improvement programs [38,39,40,41].

Among these, lncRNA-ssc.37456 exhibited distinctive spatiotemporal expression dynamics, being markedly upregulated postnatally and expressed at significantly higher levels in Tongcheng pigs than in Landrace pigs [42,43]. Such breed-specific differences are reminiscent of lncRNA-mediated modulation of muscle performance and adaptability observed across species, reflecting both the conserved and species-specific features of lncRNA function in skeletal muscle [44,45]. In a cardiotoxin (CTX)-induced muscle regeneration model, lncRNA-ssc.37456 expression was tightly correlated with the regenerative process, increasing progressively after injury and peaking at day 7—coinciding with maximal expression of the terminal differentiation marker *MYHC*. Conversely, its expression was inversely correlated with the satellite cell proliferation marker *PAX7*, indicating a potential role during the transition from proliferation to differentiation. This pattern was recapitulated in primary porcine myoblast cultures, where lncRNA-ssc.37456 levels increased concomitantly with the upregulation of *MYOG* and *MYHC* during myotube formation. These findings suggest that lncRNA-ssc.37456 acts as a differentiation-promoting regulator, functioning predominantly at late myogenic stages to facilitate the transition from proliferating myoblasts to terminally differentiated myotubes.

Functional perturbation experiments further supported this hypothesis. Knockdown of lncRNA-ssc.37456 significantly reduced *MYOG* and *MYHC* expression, impaired myotube formation, and increased EdU-positive cell ratios, indicating delayed cell cycle withdrawal and differentiation initiation. In contrast, overexpression of lncRNA-ssc.37456 enhanced myotube fusion and differentiation while suppressing proliferation. This bidirectional regulatory behavior mirrors the functions of known muscle lncRNAs such as linc-MD1, which modulates MyoD expression via a ceRNA mechanism to coordinate proliferation–differentiation balance [46,47]. The temporal and functional features of lncRNA-ssc.37456 suggest that it acts as a key regulatory node integrating proliferative arrest and terminal differentiation, possibly contributing to structural remodeling during muscle regeneration. Compared with well-characterized activation-associated lncRNAs such as PINK1-AS and SYISL, lncRNA-ssc.37456 appears to function more prominently in the differentiation phase, highlighting its potential role as a novel non-coding regulator of myogenic fate determination [48,49,50].

At the molecular level, our data provide compelling evidence that lncRNA-ssc.37456 is predominantly cytoplasmic and functions via a ceRNA mechanism to modulate post-transcriptional gene regulation. Computational prediction using the miRDB database identified 19 candidate interacting miRNAs, including miR-24-3p, miR-4437, and miR-3913-3p, several of which have established roles in myogenesis, muscle fiber type specification, and metabolic remodeling [51,52,53,54,55]. Notably, miR-24-3p regulates myoblast differentiation and muscle regeneration by targeting key myogenic factors while influencing fiber type determination and metabolic remodeling; miR-4437 has been associated with muscle growth and economically important traits in livestock, potentially regulating myoblast proliferation and differentiation; and miR-3913-3p has been linked to muscle transcriptional programs and fiber type specification in recent profiling studies [56,57,58]. Consistently, qRT-PCR analysis showed that these three miRNAs negatively regulate lncRNA-ssc.37456, and dual-luciferase reporter assays confirmed a direct interaction between miR-24-3p and lncRNA-ssc.37456, providing specific molecular evidence for this lncRNA–miRNA regulatory relationship. Collectively, these findings suggest that lncRNA-ssc.37456 functions as a multi-miRNA “sponge,” attenuating miRNA-mediated repression of downstream myogenic genes to fine-tune myoblast differentiation. This multi-miRNA ceRNA network exemplifies a mechanism by which cytoplasmic lncRNAs buffer miRNA activity to enhance both the robustness and specificity of gene expression during skeletal muscle development, representing a key regulatory strategy for myogenic differentiation and fiber type determination.

Nevertheless, several limitations should be acknowledged. First, although cross-species comparisons provide valuable references, the molecular conservation of lncRNA -ssc.37456 remains to be experimentally validated, and potential species-specific differences should be carefully considered. Second, while the differential expression of lncRNA-ssc.37456 between Tongcheng and Landrace pigs suggests a breed-dependent regulatory basis, the underlying genetic and epigenetic determinants remain elusive. Third, although we demonstrated direct binding between lncRNA-ssc.37456 and miR-26a-1, a more comprehensive delineation of its downstream targets and network-level effects is warranted. Future studies should integrate RNA immunoprecipitation (RIP), RNA pull-down, dual-luciferase, and high-throughput sequencing approaches to systematically define the lncRNA-ssc.37456–miRNA–mRNA regulatory axis. In addition, in vivo loss- and gain-of-function models combined with transcriptomic and proteomic profiling will be essential to clarify its role in fiber-type remodeling and muscle performance. Collectively, our findings uncover lncRNA-ssc. 37456 as a previously uncharacterized cytoplasmic lncRNA that promotes porcine myogenic differentiation via a ceRNA-dependent mechanism, offering new insights into the non-coding regulatory landscape of skeletal muscle development and potential molecular targets for precision breeding in livestock.

## 5. Conclusions

In summary, lncRNA-ssc.37456 is a muscle-enriched cytoplasmic lncRNA that rises during porcine myogenic differentiation and regeneration. Functional assays show that it suppresses myoblast proliferation while promoting myotube formation, acting as a key regulator of the proliferation-to-differentiation transition. Mechanistically, lncRNA-ssc.37456 functions as a ceRNA for muscle-related miRNAs (e.g., miR-24-3p) to enhance downstream myogenic gene expression. These findings identify lncRNA-ssc. 37456 as a potential molecular target for improving skeletal muscle growth and related traits in pigs.

## Figures and Tables

**Table 1:** Primers information.

Gene	Primer Sequence (5′ → 3′)	Product Length/bp
*sus-Pax7*	F: GGAGTACAAGAGGGAGAACCC	122
	R: TTCTGAGCACGCGGCTAATC	
*sus-Ki67*	F: AGCCCGTATCTGGTGCAAAA	267
	R: CCTGCATCTGTGTAAGGGCA	
*sus-MyOG*	F: AGGCTACGAGCGGACTGA;	230
	R: GCAGGGTGCTCCTCTTCA	
*sus-MyHC*	F: CTCCTGGGGTGATGGACAAC	83
	R: CTTTCTGCAGATGCGGATGC	
*sus-PCNA*	F: ATCUGGTGTGACCCGGACT	163
	R: CTGGCATCACCGAAGAGCAGTT	
*sus-GAPDH*	F: ACCCAGAAGACTGTGGATGG	79
	R: AAGCAGGGATGATGTTCTGG	

**Table 2:** Small RNA sequence.

siRNA	Sequence (5′ → 3′)
siRNA-NC	S: UUCUCCGAACGUGUCACGUTT
	A: ACGUGACACGUUCGGAGAATT
siRNA-lncRNA ssc.37456	S: GGUUCUUGGUCUCAACCAAAG
	A: UUGGUUGAGACCAAGAACCAU
miR-24-3p	UGGCUCAGUUCAGCAGGAACAG
miR-4437	UGGGCUCAGGGUACAAAGGUU
miR-3913-3p	AGACAUCAAGAUCAGUCCCAAA

S. Sense; A. Anti-sense.

## Data Availability

The data presented in this study are available from the corresponding author upon reasonable request.
